# Condition Number Estimation of Preconditioned Matrices

**DOI:** 10.1371/journal.pone.0122331

**Published:** 2015-03-27

**Authors:** Noriyuki Kushida

**Affiliations:** 1 International Data Centre, the Preparatory Commission for the Comprehensive Nuclear-Test-Ban Treaty Organization, Vienna, Austria; Cinvestav-Merida, MEXICO

## Abstract

The present paper introduces a condition number estimation method for preconditioned matrices. The newly developed method provides reasonable results, while the conventional method which is based on the Lanczos connection gives meaningless results. The Lanczos connection based method provides the condition numbers of coefficient matrices of systems of linear equations with information obtained through the preconditioned conjugate gradient method. Estimating the condition number of preconditioned matrices is sometimes important when describing the effectiveness of new preconditionerers or selecting adequate preconditioners. Operating a preconditioner on a coefficient matrix is the simplest method of estimation. However, this is not possible for large-scale computing, especially if computation is performed on distributed memory parallel computers. This is because, the preconditioned matrices become dense, even if the original matrices are sparse. Although the Lanczos connection method can be used to calculate the condition number of preconditioned matrices, it is not considered to be applicable to large-scale problems because of its weakness with respect to numerical errors. Therefore, we have developed a robust and parallelizable method based on Hager’s method. The feasibility studies are curried out for the diagonal scaling preconditioner and the SSOR preconditioner with a diagonal matrix, a tri-daigonal matrix and Pei’s matrix. As a result, the Lanczos connection method contains around 10% error in the results even with a simple problem. On the other hand, the new method contains negligible errors. In addition, the newly developed method returns reasonable solutions when the Lanczos connection method fails with Pei’s matrix, and matrices generated with the finite element method.

## Introduction

Solving the linear equation is considered to be the most time consuming component of simulation computation. As a result, a number of linear equation solvers have been developed in order to realize more efficient simulation. Linear equation solvers can be roughly categorized into two types: direct solvers and iterative solvers. Direct solvers have been used for ill conditioned problems, because of the robustness of the solution. However, the iterative method has become mainstream in recent years for the following reasons:

- The iterative method requires less memory than the direct method, if the coefficient matrix of the equation is sparse. Most simulation methods, as is the case with the finite element method and the finite difference method, use sparse matrices.
- The iterative method is more suitable for distributed memory type parallel computers than the direct method. Recently, the trend in super computers has been toward distributed memory computers.

As a result, iterative solvers have generated a great deal of interest. Iterative methods are of two types: the stationary method and the Krylov sub-space type method. Generally, the Krylov sub-space type method is more commonly used than the stationary method because the stationary method, is in most cases, slower and more suited to parallel computers. The conjugate gradient method is representative of the Krylov sub-space type method, and the Gauss-Seidel method is a typical representative example of the stationary method. The Krylov sub-space type method is faster than the stationary method, but is sometimes unstable. Furthermore, rapid convergence is always desired. In order to address such considerations, preconditioning is applied to the Krylov sub-space type method. In particular, the conjugate gradient method with preconditioning, sometimes called the preconditioned conjugate gradient method (PCG), is one of the most well known iterative solvers [1].

Evaluating the convergence rate of an iterative solver is sometimes important in order to demonstrate the effectiveness of new methods or to select an adequate method. The convergence rate of the conjugate gradient method (CG) and other Krylov sub-space type solvers strongly depends on the eigenvalue distribution of coefficient matrix, that is to say, the convergence rate is better if the eigenvalues are concentrated. Complete knowledge of the eigenvalue distribution enables the complete prediction of the convergence behavior of the PCG. However, obtaining all of the eigenvalues is difficult, and so another simple indicator, called condition number, is used. Generally, the condition number is easy to calculate when the coefficient matrix is explicitly obtained. As described above, parallel computers, especially distributed memory type parallel computers, are becoming increasingly important for engineers or physicists. Obtaining preconditioned matrices using a parallel computer is extremely difficult and an evaluation method is required. One way to evaluate preconditioned matrices is by the Lanczos connection method [2], [3]. However, the Lanczos connection method is weak with respect to round-off errors and therefore is not suitable for large matrices. Moreover, the applicability of the Lanczos connection method has not yet been discussed.

In the present paper, we introduce a new evaluation method of preconditioned matrices based on Hager’s method, and verify the robustness of the new method by comparing their precisions.

## Methods

### Preconditioning of the conjugate gradient method

#### Preconditioning

When using iterative solvers, an acceleration method called preconditioning is performed:

Let the linear equation be written as

$$\begin{array}{c} Ax=b \end{array}$$ (1)

if a preconditioning matrix **M**, which is similar to **A** in some sense, is operated on Equation 1, then Equation 1 can be written as

$$\begin{array}{c} \tilde{A}\tilde{x}=\tilde{b} \end{array}$$ (2)

where

$$\begin{array}{c} M_{1}^{-1}AM_{2}^{-1}=\tilde{A}, \end{array}$$ (3)

$$\begin{array}{c} M_{1}M_{2}=M, \end{array}$$ (4)

$$\begin{array}{c} M_{2}x=\tilde{x}, \end{array}$$ (5)

$$\begin{array}{c} M_{1}^{-1}b=\tilde{b}. \end{array}$$ (6)

In particular, the following property is required to maintain the symmetry of the preconditioned matrix:

$$\begin{array}{c} M_{1}^{T}=M_{2}. \end{array}$$ (7)

The coefficient matrix of a transformed system of linear equations (Equation 3) should be more favorable in nature than Equation 1, and the number of itrations to convergence of the CG on the transformed system should be less than that on the original system. One can show that the number of iterations to convergence of the CG is proportional to $\frac{\sqrt{\kappa }-1}{\sqrt{\kappa }+1}$, where *κ* is the 2-norm condition number of a coefficient matrix (defenition of the 2-norm condition number is given in the section “Condition number” in this article) [4]. Thus, the smaller *κ* becomes, the less iterations is required to convergence. Therefore, **M** should be selected to decrease *κ* of the transformed system. Such a transformation is not operated in practical programming, since the matrix—matrix product is needed, which requires much computational effort. The PCG algorithm is shown in Table 1. In the algorithm, Solve **Mz** = **r** is the preconditioning (line 5 in the algorithm), where **r** is the residual norm of the PCG, and **z** is the residual norm in the transformed space by **M**. Although this equation is usually difficult to solve, such difficulty can be avoided by selecting matrix **M** to be a diagonal matrix or products of triangular matrices. Such matrices are described in the following two sections.

**Table 1:** Preconditioned Conjugte Gradient Algorithm.

1:	**procedure** PCG(**A**, **b**)
2:	Let **x**_0_ be the initial approximation
3:	**r**_0_ = **b** − **Ax**_0_
4:	**for** *i* = 0, 1, 2, 3, …, until convergence do **do**
5:	solve **Mz**_*i*_ = **r**_*i*_
6:	*ρ*_*i*_ = ⟨**r**_*i*_, **z**_*i*_⟩
7:	**If** *i* = 0 **then**
8:	**p**_*i*+1_ = **z**_*i*_
9:	**else**
10:	$\beta_{i}=\frac{\rho_{i}}{\rho_{i-1}}$
11:	**p**_*i*+1_ = **z**_*i*_ + *β*_*i*_ **p**_*i*_
12:	**end if**
13:	**q**_*i*+1_ = **A** **p**_*i*+1_
14:	$\alpha_{i}=\frac{\rho_{i}}{\left\langle {\text{p}}_{i+1},{\text{q}}_{i+1}\right\rangle }$
15:	**x**_*i*+1_ = **x**_*i*_ + *α*_*i*_ **p**_*i*+1_
16:	**r**_*i*+1_ = **r**_*i*_ + *α*_*i*_ **q**_*i*+1_
17:	**end for**
18:	return **x**_*i*_*last*__, where *i*_*last*_ is the last iteration
19:	**end procedure**

Pseudocode of the PCG. In the algorithm,⟨⋅, ⋅⟩ denotes vector inner product.

### Diagonal scaling

When the preconditioning matrix **M** is expressed as

$$\begin{array}{c} M=diag\;\left(A\right), \end{array}$$ (8)

where the function *diag*(**A**) extract the diagonal component of a matrix **A**. This preconditioning is called the diagonal scaling [5].

### SSOR

Assuming that the coefficient matrix is symmetric and is decomposed as

$$\begin{array}{c} A=D+L+L^{T} \end{array}$$ (9)

the SSOR preconditioning matrix is defined as

$$\begin{array}{c} M\left(\omega \right)=\frac{1}{2-\omega }\left(\frac{1}{\omega }D+L\right)\;{\left(\frac{1}{\omega }D\right)}^{-1}\;{\left(\frac{1}{\omega }D+L\right)}^{T} \end{array}$$ (10)

where, **D**, **L**, and *ω* are the diagonal component of **A**, the lower triangular component of **A**, and the relaxation parameter, respectively. Usually *ω* is set to 1.0 [6]. Note that the diagonal scaling and SSOR preconditioning matrices are symmetric positive definite (SPD), if **A** is SPD. This is because, those preconditioning matrices can be rewritten as,

$$\begin{array}{c} M=\hat{L}{\hat{L}}^{T}, \end{array}$$ (11)

where $\hat{\text{L}}$ is a solvable lower triangular matrix, and then, Equation 11 is the definition of the Cholesky decomposition. Thus **M** is SPD [7], [8].

### Condition number

The convergence rate of the CG method depends on the distribution of eigenvalues of a coefficient matrix **A** or a preconditioned matrix $\tilde{\text{A}}$. Although the complete information of eigenvalues enables prediction of the exact convergence behavior, a simpler indicator, known as the condition number, exists. The condition number of a matrix is given by

$$\begin{array}{c} cond\left(A\right)=∥A∥∥A^{-1}∥ \end{array}$$ (12)

where ‖⋅‖ denotes the norm of a matrix [8]. Since there are various definitions of the norm, we can define various condition numbers. The condition numbers given by the 1-norm or 2-norm, as defined below, are commonly used.

$$\begin{array}{c} {∥A∥}_{1}=\max_{j}\sum_{i=1}^{n}\left|a_{ij}\right| \end{array}$$ (13)

$$\begin{array}{c} {∥A∥}_{2}=\left(\sqrt{maximum\;eigenvalue\;ofA^{T}A}\right) \end{array}$$ (14)

Furthermore, if the matrix **A** is SPD, then the condition number given by the 2-norm can be written as

$$\begin{array}{c} cond\left(A\right)=\frac{maximum\;eigenvalue\;of\;A}{minimum\;eigenvalue\;of\;A} \end{array}$$ (15)

### Lanczos connection

The Lanczos connection method is a method of solving the eigensystem and is strongly related to the CG method [2]. Using such a relationship, the condition number of **A** can be calculated through the CG procedure. Now, we define the *n* × *k* matrix **R**_*k*_ as

$$\begin{array}{c} R_{k}=\left[r_{0},r_{1},\ldots ,r_{k-1}\right] \end{array}$$ (16)

where *n* is the matrix size of **A**, *k* is the iteration number of the PCG and **r**_*i*_, (*i* = 0, …, *k* − 1) are the residual vectors and can be found in the PCG algorithm (Table 1).

Next, we define *k* × *k* matrix **B**_*k*_ as follows:

$$\begin{array}{c} B_{k}=\left[\begin{array}{ccccc} 1 & -\beta_{1} & & \cdots & 0\\ & 1 & -\beta_{2} & & \vdots \\ & & \ddots & \ddots & \\ & & & \ddots & -\beta_{k-1}\\ & & & & 1 \end{array}\right], \end{array}$$ (17)

where *β*_*k*_ are the scalars shown in the PCG algorithm. Using the matrix **P**_*k*_ = [**p**_0_, **p**_1_, …, **p**_*k*−1_],

$$\begin{array}{c} R_{k}=P_{k}B_{k}. \end{array}$$ (18)

where **p**_*i*_, (*i* = 0, …, *k* − 1) are the modification direction vectors in the PCG algorithm. Since vectors **p**_1_, **p**_2_, …, **p**_*k*_ are **A**-orthogonal,

$$\begin{array}{c} {\hat{T}}_{k}=R_{k}^{T}AR_{k}=B_{k}^{T}\Lambda_{k}B_{k}, \end{array}$$ (19)

where Λ_*k*_ is a matrix having components that are given by

$$\begin{array}{c} \Lambda_{k}=\left[\begin{array}{ccccc} p_{1}^{T}Ap_{1} & & & \\ & p_{2}^{T}Ap_{2} & & & \\ & & & \ddots & \\ & & & & p_{k}^{T}Ap_{k} \end{array}\right]. \end{array}$$ (20)

Here, we consider the *n* × *k* matrix **Q**_*k*_,

$$\begin{array}{c} Q_{k}=R_{k}\Delta_{k}^{-1}. \end{array}$$ (21)

where Δ_*k*_ is a *k* × *k* matrix having the following components:

$$\begin{array}{c} \Delta_{k}=\left[\begin{array}{cccc} {∥r_{1}∥}_{2} & & & \\ & {∥r_{2}∥}_{2} & & \\ & & \ddots & \\ & & & {∥r_{k}∥}_{2} \end{array}\right], \end{array}$$ (22)

where ‖**r**_*i*_‖_2_, (*i* = 0, …, *k*) are the 2-norms of the residual vectors. The columns of **Q**_*k*_ are the Lanczos vectors, for which the associated projections of **A** form a tridiagonal matrix, thus

$$\begin{array}{c} T_{k}=\Delta_{k}^{-1}B_{k}^{T}\Lambda_{k}B_{k}\Delta_{k}^{-1}. \end{array}$$ (23)

Since external eigenvalues of **T**_*k*_ approximate those of matrix **A**, we can approximate the condition number of matrix **A** using this method. Furthermore, if the above parameters are obtained from the PCG, we can obtain the condition number of the preconditioned matrix [3]. The advantages of this method are ease of implementation and ease of evaluating the condition number of preconditioned matrices. However, this method is not applicable for large systems or ill-conditioned systems, due to the fact that this method requires the strict orthogonality of basis vector used in the CG aglgorithm. However, such orthogonality tends to be broken for the large system or ill conditioned system.

### Hager’s method

Hager’s method gives the 1-norm of **A** ^−1^ [9]. Originally, the condition number is defined as Equation 12. Therefore, we need the 1-norm of **A**. Fortunately, the 1-norm of **A** is easy to obtain if the components of **A** are given explicitly. The algorithm of Hager’s method is described in Table 2. Although Hager’s method requires the transposition of matrix **A**, we assume that **A** ^*T*^ = **A**, because we are using the PCG, therefore symmetric positive definite matrices can be solved. Hager’s method requires the solution of a linear equation during iteration of the procedure for finding the 1-norm condition number. In the present study, they are solved using the PCG.

**Table 2:** The algorithm of Hager’s method.

1:	**procedure** Hager’s method(**A**)
2:	Set $\text{b}={\left(\frac{1}{n},\cdots ,\frac{1}{n}\right)}^{T}$, where *n* is the size of the matrix **A**, and *ρ* = 0
3:	**for do**
4:	Solve **Ax** = **b**
5:	**if** ‖**x**‖_1_ ≤ *ρ* **then**
6:	return *ρ* as ‖**A** ^−1^‖_1_, and exit
7:	**else**
8:	*ρ* = ‖**x**‖_1_
9:	**end if**
10:	**y**[*i*] = sgn (**x**[*i*]) (*i* = 1, 2, …, *n*)
11:	Solve **A** ^*T*^ **z** = **y**
12:	*i*_max_ = *i* which satisfies $\underset{i}{\text{max}}\;\left|z[i]\right|$
13:	**if** ∣**z**[*i*_*max*_]∣ < **z** ^*T*^ **b** **then**
14:	return *ρ* as ‖**A** ^−1^‖_1_, and exit
15:	**else**
16:	$b[i]=\{\begin{array}{c} 1\;\;\left(i=i_{\max}\right)\\ 0\;\;\left(i\neq i_{\max}\right) \end{array}$
17:	**end if**
18:	**end for**
19:	**end procedure**

Pseudocode of Hager’s method. In the algorithm, [*i*] denotes the *i*th vector element of a vector.

## Hager’s method for preconditioned matrices

Here, we develop a new condition number estimation method which overcomes the downside of the Lanczos connection method. That is to say, new method must have such features:

- Applicable for large system. Especially, it must work on distributed memory computers.
- Robust for ill conditioned system.

In order to develop such new method, we employ Hager’s method which gives the 1-norm of inverse matrices as a basic algorithm. In the rest of this sections, we describe the algorithm of our new method.

As introduced in the previous section, we can obtain the 1-norm condition number using Hager’s method. There is a simple method of extending Hager’s method to preconditioned matrices without explicit matrix production. That is, in Hager’s method, we simply replace matrix **A** with ${\text{M}}_{1}^{-1}\text{A}{\text{M}}_{2}^{-1}$. It can be easily achieved if the PCG is employed to solve equations that appear in Hager’s method (lines 4 and 11 in Table 2). This is because, matrices are used as the form of **p** = **Aq**, where **p** and **q** are vectors. Therefore, we can solve the system of ${\text{M}}_{1}^{-1}\text{A}{\text{M}}_{2}^{-1}\text{x}=\text{b}$ by replacing **A** with ${\text{M}}_{1}^{-1}\text{A}{\text{M}}_{2}^{-1}$ in the PCG algorithm. The algorithm is named “calc 1-norm of inverse preconditioned matrix” and is shown in Table 3. In addition, the PCG algorithm that solves ${\text{M}}_{1}^{-1}\text{A}{\text{M}}_{2}^{-1}\text{x}=\text{b}$ is shown in Table 4.

**Table 3:** The algorithm of the modified Hager’s method.

1:	**procedure** Modified Hager’s method (${\text{M}}_{1}^{-1}$, **A**, ${\text{M}}_{2}^{-1}$)
2:	*ρ*_*inv*_ = call procedure calc 1-norm of inverse preconditioned matrix (${\text{M}}_{1}^{-1}$, **A**, ${\text{M}}_{2}^{-1}$)
3:	*ρ*_*forward*_ = call procedure calc 1-norm of preconditioned matrix(${\text{M}}_{1}^{-1}$, **A**, ${\text{M}}_{2}^{-1}$)
4:	return *ρ*_*inv*_ ⋅ *ρ*_*forward*_ as the condition number of ${\text{M}}_{1}^{-1}{\text{AM}}_{2}^{-1}$
5:	**end procedure**
6:	**procedure** calc 1-norm of inverse preconditioned matrix (${\text{M}}_{1}^{-1}$, **A**, ${\text{M}}_{2}^{-1}$)
7:	Set $\text{b}={\left(\frac{1}{n},\cdots ,\frac{1}{n}\right)}^{T}$ and *ρ*_*inv*_ = 0
8:	**for do**
9:	**x** call procedure PCG in modified Hager (${\text{M}}_{1}^{-1}$, **A**, ${\text{M}}_{2}^{-1}$, **b**)
10:	**if** ‖**x**‖_1_ ≤ *ρ*_*inv*_ **then**
11:	return *ρ*_*inv*_ as $\|{\left({\text{M}}_{1}^{-1}{\text{AM}}_{2}^{-1}\right)}^{-1}{\|}_{1}$, and exit
12:	**else**
13:	*ρ*_*inv*_ = ‖**x**‖_1_
14:	**end if**
15:	**y**[*i*] = sgn (**x**[*i*]) (*i* = 1, 2, …, *n*)
16:	**z** = call procedure PCG in modified Hager (${\text{M}}_{1}^{-1}$, **A**, ${\text{M}}_{2}^{-1}$, **y**)
17:	*i*_max_ = *i* which satisfies $\underset{i}{\text{max}}\;\left|z[i]\right|$
18:	**if** ∣**z**[*i*_*max*_]∣ < **z** ^*T*^ **b** **then**
19:	return *ρ*_*inv*_ as $\|{\left({\text{M}}_{1}^{-1}{\text{AM}}_{2}^{-1}\right)}^{-1}{\|}_{1}$, and exit
20:	**else**
21:	$\text{b}[i]=\{\begin{array}{c} 1\;\;\left(i=i_{\max}\right)\\ 0\;\;\left(i\neq i_{\max}\right) \end{array}$
22:	**end if**
23:	**end for**
24:	**end procedure**
25:	**procedure** calc 1-norm of preconditioned matrix (${\text{M}}_{1}^{-1}$, **A**, ${\text{M}}_{2}^{-1}$)
26:	Set $\text{b}={\left(\frac{1}{n},\cdots ,\frac{1}{n}\right)}^{T}$ and *ρ*_*forwad*_ = 0
27:	**for do**
28:	$\text{x}={\text{M}}_{1}^{-1}\text{A}{\text{M}}_{2}^{-1}\text{b}$
29:	**if** ‖**x**‖_1_ ≤ *ρ*_*forward*_ **then**
30:	return *ρ*_*forward*_ as $\|{\text{M}}_{1}^{-1}{\text{AM}}_{2}^{-1}{\|}_{1}$, and exit
31:	**else**
32:	*ρ*_*forward*_ = ‖**x**‖_1_
33:	**end if**
34:	**y**[*i*] = sgn (**x**[*i*]) (*i* = 1, 2, …, *n*)
35:	$\text{z}={\text{M}}_{1}^{-1}\text{A}{\text{M}}_{2}^{-1}\text{y}$
36:	*i*_max_ = *i* which satisfies $\underset{i}{\text{max}}\;\left|z[i]\right|$
37:	**if** ∣**z**[*i*_*max*_]∣ < **z** ^*T*^ **b** **then**
38:	return *ρ*_*forward*_ as $\|{\text{M}}_{1}^{-1}{\text{AM}}_{2}^{-1}{\|}_{1}$, and exit
39:	**else**
40:	$\text{b}[i]=\{\begin{array}{c} 1\;\;\left(i=i_{\max}\right)\\ 0\;\;\left(i\neq i_{\max}\right) \end{array}$
41:	**end if**
42:	**end for**
43:	**end procedure**

Pseudocode of the modified Hager’s method. The modified Hager’s method consists of two major parts: one is for calculating $\|{\left({\text{M}}_{1}^{-1}{\text{AM}}_{2}^{-1}\right)}^{-1}{\|}_{1}$ and the other is for $\|{\text{M}}_{1}^{-1}{\text{AM}}_{2}^{-1}{\|}_{1}$. In the algorithm, [*i*] denotes the *i*th vector element of a vector.

**Table 4:** Preconditioned Conjugte Gradient Algorithm in the modified Hager’s method.

1:	**procedure** PCG in modified Hager (${\text{M}}_{1}^{-1}$, **A**, ${\text{M}}_{2}^{-1}$, **b**)
2:	Let **x**_0_ be the initial approximation
3:	${\text{r}}_{0}=\text{b}-{\text{M}}_{1}^{-1}\text{A}{\text{M}}_{2}^{-1}{\text{x}}_{0}$
4:	**for** *i* = 0, 1, 2, 3, …, until convergence do **do**
5:	*ρ*_*i*_ = ⟨**r**_*i*_, **r**_*i*_⟩
6:	**if** *i* = 0 **then**
7:	**p**_*i*+1_ = **r**_*i*_
8:	**else**
9:	$\beta_{i}=\frac{\rho_{i}}{\rho_{i-1}}$
10:	**p**_*i*+1_ = **r**_*i*_ + *β*_*i*_ **p**_*i*_
11:	**end if**
12:	${\text{q}}_{i+1}={\text{M}}_{1}^{-1}\text{A}{\text{M}}_{2}^{-1}{\text{p}}_{i+1}$
13:	$\alpha_{i}=\frac{\rho_{i}}{\left\langle {\text{p}}_{i+1},{\text{q}}_{i+1}\right\rangle }$
14:	**x**_*i*+1_ = **x**_*i*_ + *α*_*i*_ **p**_*i*+1_
15:	**r**_*i*+1_ = **r**_*i*_ + *α*_*i*_ **q**_*i*+1_
16:	**end for**
17:	return **x**_*i*_*last*__, where *i*_*last*_ is the last iteration
18:	**end procedure**

Pseudocode of the PCG in the modified Hager’s method. In the algorithm,⟨⋅, ⋅⟩ denotes vector inner product.

Since the Hager’s method gives us only $\|{\left({\text{M}}_{1}^{-1}\text{A}{\text{M}}_{2}^{-1}\right)}^{-1}{\|}_{1}$, a method by which to calculate $\|{\text{M}}_{1}^{-1}\text{A}{\text{M}}_{2}^{-1}{\|}_{1}$ must be constructed. It is not straightforward, because we have no explicit ${\text{M}}_{1}^{-1}\text{A}{\text{M}}_{2}^{-1}$. In the present study, we develop such a method using Hager’s method by replacing the solution of the two linear equations in Table 2 (lines 4 and 11) with ${\text{M}}_{1}^{-1}\text{A}{\text{M}}_{2}^{-1}\text{b}=\text{x}$ and ${\text{M}}_{1}^{-1}\text{A}{\text{M}}_{2}^{-1}\text{y}=\text{z}$, respectively. We thus obtain $\|{\text{M}}_{1}^{-1}\text{A}{\text{M}}_{2}^{-1}{\|}_{1}$. The algorithm which calculates the 1-norm of preconditioned matrix is named “calc 1-norm of preconditioned matrix” and is shown in Table 3. The entire algorithm to calculate 1-norm condition number is referred to as the modified Hager’s method in the present article.

These developed algorithms for preconditioned matrices always converge if coefficient matrices and preconditioning matrices are positive definite. This is because, Hager’s method always converges for positive definite matrices [10]. In addition, one can show that the inverse matrices of positive definite matrices are also positive definite, and the product of two positive definite matrices are also positive definite.

## Results

In this section, we carry out the feasibility test of our new method by comparing the performance of both our method and the Lanczos connection method. In the following sections, the computational environment, and the matrices which we employ will be explained, and the performances are also described.

### Computational environment

Here, we describe the computational environment in order to determine the precision of the numerical calculation. All of the code was written with Fortran90, and the eigenvalues of the tridiagonal matrix obtained from the Lanczos connection was calculated using the Intel Math Kernel library. The machine architecture and compiler environment are listed in Table 5. In addition, the condition number, which should be compared between the Lanczos connection method and our modified Hager’s method, was calculated using Octave [11].

**Table 5:** Computational Environment.

Architecture	Intel Pentium4 2.8 GHz
Operating System	Vine linux 3.1
Compiler	Intel Fortran Compiler 8.1
Compiler options	-O3
Math library	Intel Math Kernel library
Float number precision	real(8)

Details of the computational environment used for the precision verification.

### Sample matrices

#### Diagonal matrix

In order to verify the precision of both of these methods, we first employ a simple diagonal matrix for a test problem. The test matrix **T**_*diag*_ is given by

$$\begin{array}{c} T_{diag}=\left[\begin{array}{cccc} 1 & & & \\ & 2 & & \\ & & \ddots & \\ & & & n \end{array}\right], \end{array}$$ (24)

where *n* is the matrix size. The eigenvalues of this matrix are (1, 2, …, *n*), and the condition number of these eigenvalues should be *n*. Note that, the condition number of both diagonal scaled and SSOR preconditioned **T**_*diag*_ are 1.0.

#### Tridiagonal matrix

The *n* × *n* tridiagonal matrix we employed in the present studies is given as

$$\begin{array}{c} T_{Tri}=\left[\begin{array}{cccc} 2 & -1 & & \\ -1 & 2 & \ddots & \\ & \ddots & \ddots & -1\\ & & -1 & 2 \end{array}\right]. \end{array}$$ (25)

#### Pei’s matrix

We employ Pei’s matrix [12] as a test matrix, because one can control the condition number of the matrix quite easily. Pei’s matrix is defined as follows:

$$\begin{array}{c} T_{Pei}=\left[\begin{array}{ccccc} 1+d & 1 & \cdots & \cdots & 1\\ 1 & 1+d & 1 & \cdots & 1\\ \vdots & 1 & 1+d & \ddots & 1\\ \vdots & \vdots & \ddots & \ddots & 1\\ 1 & 1 & 1 & 1 & 1+d \end{array}\right], \end{array}$$ (26)

where *d* is the parameter by which to determine the condition of matrix **T**_*Pei*_, which becomes increasingly ill conditioned as *d* approaches zero. If *d* is zero, matrix **T**_*Pei*_ is singular.

#### Poission’s equation with FEM

The above sample matrices were artificially created and have simple structures. In this section, we will introduce matrices that are created with the finite element method (FEM) in order to investigate the feasibility of both methods with realistic problems. The equation which employed was Poission’s equation. The analysis domain was a cube with edge lengths of 1.0. In the present study, three types of finite element meshes were prepared and each of three types had a different degree of freedom (DOF) on *x*, *y*, and *z* axes: 20 × 20 × 20, 10 × 10 × 80, and 5 × 5 × 320. The total DOFs were the same. Dirichlet boundary conditions were applied to both faces at *z* = 0, and 1.0.

### Estimated condition numbers

#### Diagonal matrix

First we discuss the feasibility of the Lanczos connection. The eigenvalues of **T**_*diag*_ calculated with the Lanczos connection is listed in the first row in Table 6. Here we set *n* = 10. The largest eigenvalue is unreasoably large, and therefore, obtained condition number is unreasonable. We can remove this unresonably large eigenvalue, by ignoring the first step information of the PCG. This treatment leads one-rank smaller tri-diagonal matrix associated with the Lanczos connection. However, the resulted condition number is more reasonable. The eigenvalues without the first step information is list in the second row in Table 6. Simply, the largest eigenvalue is removed. This treatment is applied to the remaining of this article.

**Table 6:** Eigenvalues of a 10 × 10 diagonal matrix calculated using the Lanczos connection method.

Ideal	1	2	3	4	5	6	7	8	9	10
Lanczos connection	1.00	2.02	3.04	4.08	5.13	6.17	7.28	8.37	9.51	3.0×10^6^
Without 1st step	1.00	2.02	3.04	4.08	5.13	6.20	7.28	8.37	9.51	NA

In the table, the eigenvalues of a 10 × 10 diagonal matrix calculated using the Lanczos connection method are listed. The first row indicates the eigenvalues with all information, and the second row indicates the eigenvalues without the first CG step information. In the full information case, the condition number becomes unreasonable, while the second one shows a reasonable result. In the table, NA means “Not Available”.

Next, we check the feasibility of both the Lanczos connection and Hager’s method with **T**_*diag*_. The condition numbers calculated using both methids are listed in Table 7. The obtained condition numbers are reasonable.

**Table 7:** Condition numbers of **T**_*diag*_ by the Lanczos connection method and Hager’s method.

Matrix Size	10	100	200	500
Lanczos connection	9.49	99.74	199.78	499.81
Octave (2-norm cond. num.)	10	100	200	500
Hager’s method	10.00	100.00	200.00	500.00
Octave (1-norm cond. num.)	10	100	200	500

In the table, condition numbers calculated using both the Lanczos connection method and Hager’s method are listed with numbers with Octave. Both method show good performances, but the Lanczos method includes some error, although the test matrix is simple.

#### Tridiagonal matrix

The condition numbers of **T**_*Tri*_ are listed in Table 8. Here we employ 10 × 10, 100 × 100, 200 × 200 and 500 × 500 matrices. In addition, those of diagonally scaled matrices of the same size are listed in Table 9. Comparison of Table 8. Table 9 reveals that the condition number is not improved by diagonal scaling in this case. Moreover, the number of iterations to convergence was also the same. We first discuss Hager’s method, which corresponds to Octave completely, for every size in both cases. Higham reported that Hager’s method gives the correct value for positive definite matrices [10]. As we discussed, the diagonal scaled matrices are also SPD. Therefore, these results can be expected. On the other hand, although the Lanczos connection method provides less favorable results for smaller matrices, the results improved for larger problems. This behavior results from the modification described in the previous section, whereby we removed the information of the first conjugate gradient step. The effects of the modification became smaller for larger problems. Finally, it can be said that software developed in the present studies of both the Lanczos connection method and the modified Hager’s method work well with preconditioned matrices.

**Table 8:** Condition numbers: Tridiagonal matrix.

Matrix Size	10	100	200	500
Lanczos connection	41.8	4.13 × 10^3^	1.64 × 10^4^	1.02 × 10^5^
Octave (2-norm cond. num.)	48.0	4.13 × 10^3^	1.64 × 10^4^	1.02 × 10^5^
Hager’s method	60.0	5.10 × 10^3^	2.02 × 10^6^	1.26 × 10^6^
Octave (1-norm cond. num.)	60.0	5.10 × 10^3^	2.02 × 10^6^	1.26 × 10^6^

The condition numbers of **T**_*Tri*_ using the Lanczos connection method, the modified Hager’s method, and Octave are listed.

**Table 9:** Condition numbers: Diagonal scaled tridiagonal matrix.

Matrix Size	10	100	200	500
Lanczos connection	41.8	4.13 × 10^3^	1.64 × 10^4^	1.02 × 10^5^
Octave (2-norm cond. num.)	48.0	4.13 × 10^3^	1.64 × 10^4^	1.02 × 10^5^
Modified Hager’s method	60.0	5.10 × 10^3^	2.02 × 10^6^	1.26 × 10^6^
Octave (1-norm cond. num.)	60.0	5.10 × 10^3^	2.02 × 10^6^	1.26 × 10^6^

The condition numbers of diagonal scaled **T**_*Tri*_ using the Lanczos connection method, the modified Hager’s method, and Octave are listed.

#### Pei’s matrix

In the present paper, we fixed the matrix size as 100 × 100 and set *d* as *d* = 0.5, 0.25 and 0.125. Calculated condition numbers and errors with respect to the condition number from Octave are listed in Table 10. An error is defined as

$$\begin{array}{c} Error=\frac{\left|\mathrm{Calculated}\;\;\mathrm{condition}\;\;\mathrm{number}-\mathrm{Octave}\right|}{\mathrm{Octave}}\times 100\left(\%\right). \end{array}$$ (27)

The condition numbers estimated by the Lanczos connection method are 64,851(*d* = 0.5), 178,699(*d* = 0.25) and 338,463(*d* = 0.125), and those estimated by Octave are 1,365.6(*d* = 0.5), 3,259.8(*d* = 0.25) and 7,224.7(*d* = 0.125). Thus, in the Lanczos connection method case, the error is always larger than 4,000%, and the results diverge greatly from the correct answers. Because the Lanczos connection showed the reasonable results on SSOR preconditioned matrices in the article [3], it can be considered that these results are caused by the weakness with respect to numerical errors of the CG method, and great care in usage is required. On the other hand, the condition numbers estimated by the modified Hager’s method are 1,684.08(*d* = 0.5), 4,020.75(*d* = 0.25) and 8,911.86(*d* = 0.125), and those estimated by Octave 1,684.1(*d* = 0.5), 4,020.8(*d* = 0.25) and 8,911.9(*d* = 0.125). That is to say, the modified Hager’s method provided the best results for all cases.

**Table 10:** Condition numbers: SSOR preconditioned Pei’s matrix for various *d*.

	Lanczos	Octave	Error(%)	Modified Hager	Octave	Error (%)
*d* = 0.5	64,851	1,365.6	4,648.9	1,684.08	1,684.1	0.0012
*d* = 0.25	178,699	3,259.8	5,387.0	4,020.75	4,020.8	0.0012
*d* = 0.125	338,463	7,224.7	4,584.8	8,911.86	8,911.9	0.0004

The condition numbers of SSOR preconditioned **T**_*Pei*_ using the Lanczos connection method, the modified Hager’s method, and Octave are listed. In this table, error values defined as $Error=\frac{|\text{Calculated}\;\;\text{condition}\;\;\text{number}-\text{Octave}|}{\text{Octave}}\times 100(\%)$ are also indicated.

#### Poission’s equation with FEM

The condition numbers of preconditioned matrices of Poisson’s equation are listed in Table 11, and 12. The errors are also shown in the tables. In diagonal scaled cases, the Lanczos connection gives results with over 1,000% errors. Particularly, in the 20 × 20 × 20 case, it shows 3.03 × 10^27^% error. Reasonable results can be obtained by removing the last CG step information from the Lanczos connection procedure. The condition number moves from 2.35 × 10^28^ to 6.94 × 10^2^. However, such procedures can only be done when the true result is already known, and therefore, the Lanczos connection cannot be used with ease. On the other hand, using the modified Hager’s method gives the results with an error rate under 6.46%.

**Table 11:** Condition numbers: Diagonal scaled Poisson–FEM matrix.

	Lanczos	Octave	Error(%)	Modified Hager	Octave	Error(%)
20 × 20 × 20	2.35 × 10^28^	7.75 × 10^2^	3.03 × 10^27^	2.98 × 10^2^	2.98 × 10^2^	0.00
10 × 10 × 80	5.82 × 10^4^	2.79 × 10^3^	1.98 × 10^3^	3.63 × 10^3^	3.87 × 10^3^	6.43
5 × 5 × 320	5.98 × 10^5^	4.73 × 10^4^	1.16 × 10^3^	6.16 × 10^4^	6.59 × 10^4^	6.46

The condition numbers of the diagonal scaled Poisson–FEM matrices using the Lanczos connection method, the modified Hager’s method, and Octave are listed. In this table, error values defined as $Error=\frac{|\text{Calculated}\;\;\text{condition}\;\;\text{number}-\text{Octave}|}{\text{Octave}}\times 100(\%)$ are also indicated.

**Table 12:** Condition numbers: SSOR preconditioned Poisson–FEM matrix.

	Lanczos	Octave	Error(%)	Modified Hager	Octave	Error(%)
20 × 20 × 20	4.12 × 10^2^	8.22 × 10^2^	49.84	1.05 × 10^3^	1.07 × 10^3^	2.37
10 × 10 × 80	4.82 × 10^2^	2.47 × 10^3^	80.49	8.48 × 10^3^	8.53 × 10^3^	0.64
5 × 5 × 320	1.22 × 10^5^	7.61 × 10^5^	84.03	1.50 × 10^6^	1.50 × 10^6^	0.00

The condition numbers of SSOR preconditioned Poisson–FEM matrices using the Lanczos connection method, the modified Hager’s method, and Octave are listed. In this table, error values defined as $Error=\frac{|\text{Calculated}\;\;\text{condition}\;\;\text{number}-\text{Octave}|}{\text{Octave}}\times 100(\%)$ are also indicated.

In SSOR preconditioned cases, the Lanczos connection gives more accurate results than diagonal scaled cases, however, the results still include over 50% errors. In addition, when the condition number of the original matrix becomes large, the error becomes more extreme. Estimations may become unreasonable in cases where problems are ill-conditioned. The modified Hager’s method gives results with under 3% errors. The results in this section show that the modified Hager’s method can be used to estimate the condition numbers of practical problems. Finally, for reference, the condition numbers of each problem based on each norm without preconditioning are indicated below: 1-norm 20 × 20 × 20: 1.02 × 10^3^, 10 × 10 × 80: 3.73 × 10^3^, and 5 × 5 × 320: 7.22 × 10^4^, 2-norm 20 × 20 × 20: 7.75 × 10^2^, 10 × 10 × 80: 2.79 × 10^3^, and 5 × 5 × 320: 4.73 × 10^4^. The condition numbers without preconditioning were calculated with Octave. Sample programs of both the Lanczos connection and the modified Hager’s method will be uploaded on the Zenodo site for readers’ convenience [13].

## Discussion

### Parallel implementation

In this section, the research examines how to implement the PCG, and the modified Hager’s method. First, the algorithm of the PCG (Table 1) is considered. The PCG algorithm consists of just three components: (1) a matrix–vector product (lines 3, and 13), (2) a vector inner product (lines 6, and 14), and (3) a scalar–vector product (lines 11, 15, and 16). By taking into account the above point, the PCG on distributed memory parallel computers is usually implemented with row-wise matrix decompoistion manner [6], [14]. Here we assume that there are two processors, an 8 × 8 matrix, and an 8 elements vector. The first processor has rows from one to four of the matrix, and vector elements from one to four. The other processor has the remaining matrix rows and vector elements. This memory allocation enables a user to compute all the above three operations with minimal (or even zero) communication, which deteriorates parallel efficiency. Namely, (1) matrix–vector products can be performed if necessary vector elements are obtained, (2) vector inner products can be performed by exchanging the results of partial vector inner products (which are just scalars) on each processor, and (3) scalar–vector products can be performed without any communication. One drawback of this memory allocation is that some preconditioning techniques, like SSOR, cannot be implemented on parallel computers as they are on sequential computers. One of the ways to accomplish such a preconditioning technique is localization, which uses only the information that is on each processor, and ignores the information on the other processors [6]. This ignoring of information degrades the effect of preconditioning; however the entire PCG algorithm still works successfully.

Hager’s method, and therefore the modified Hager’s method as well, can be implemented with the memory allocation techniques explained above. There are several additional operations to PCG (e.g. lines 10, 12, and 16 in Table 2), but they can be implemented without any difficulties. In addition, it should be noted that there is no extra memory space required for parallel computing, except the vector elements which must be obtained from other processors.

Finally, the parallel version of the modified Hager’s method was implemented with the above technique. In addition, it was confirmed that the same results as the sequential version were obtained with the diagonal scaling preconditioning.

### Computational effort and memory usage

Here, the research discusses the computational effort and the memory usage of the Lanczos connection, the modified Hager’s method, and the naive implementation that computes preconditioned matrices explicitely. First, the computational effort, and the memory usage of the naive implementation are proportional to *n* ^3^, and *n* ^2^ respectively, where *n* is the size of a matrix. Here, we assume that the Householder transformation is used to calculate the eigenvalues. Thus, both the computational effort and the memory usage are colossal when *n* is large and it is applicable only when *n* is sufficiently small.

Second, the computational effort and the memory usage of the Lanczos connection are proportional to *m* × *n* and *n* respectively, where *m* is the number of iterations required to achieve convergence of the PCG algorithm. Here, we assume that the matrix we consider has the same matrix structure as the matrices used in the section “Poisson’s equation with FEM”. In addition, we can assume that *m* < < *n*, in other words, the tridiagonal matrix associated with the Lanczos connection is small and the computational effort required to solve is negligible. The matrix has 10 matrix elements in each row, therefore, the entire size becomes 10 × *n*. The PCG algorithm requires six vectors. In the end, 16 × *n* elements must be stored. The number of floating point operations of an iteration of the PCG algorithm is approximately twice (add and multiply) the number of matrix and vector elements (excluding preconditioning). The diagonal scaling requries *n* floating point operations, and the SSOR preconditioning requries 20 × *n* floating point operations. As a result, roughly 50 × *m* × *n* floating point operations are required.

Finally, we consider the modified Hager’s method. The memory space requirement of the modified Hager’s method is almost the same as the PCG, but additional four vectors must be stored. Experiments in this study indicate that Hager’s method converges within four iterations. In addition, Higham also reported that Hager’s method converges within four iterations [10]. Using Hager’s method, solving linear equations requires the most computational effort and must be done twice par iteration. Other operations, however, require negligible effort. Thus, the total number of floating point operations can be written as 4 iterations ×2 PCGs ×50 × *m* × *n*. The computational effort required of the modified Hager’s method can be as much as ten times larger than effort required of the Lanczos connection. Nevertheless, the memory usage required for each method is almost identical, and the modified Hager’s method is more reliable than the Lanczos connection.

## Conclusions

In present paper, we developed a condition number estimation method for the preconditioned matrix and verified the accuracy of the newly developed method. Although the Lanczos connection method can be considered as a method by which to estimate the condition number of the preconditioned matrix, this method is weak with respect to numerical errors. Therefore, we compared the condition numbers estimated using both the Lanczos connection method and the newly developed method with Octave’s result. The newly developed method provided better results, whereas the Lanczos connection method failed. In addition, the newly developed method can be applied without difficulty for parallel computing and large-scale problem because this new method does not require explicit matrix operation.
