# Correction: Condition Number Estimation of Preconditioned Matrices

**DOI:** 10.1371/journal.pone.0130920

**Published:** 2015-06-17

**Authors:** Noriyuki Kushida

There is an additional affiliation for the first author. Noriyuki Kushida is also affiliated with: Center for Computational Science and E-systems, Japan Atomic Energy Agency, Ibaraki, Japan
